# Two new species of *Monodontomerus* (Hymenoptera, Torymidae) from the Pacific Northwest of North America and a revised key to the genus

**DOI:** 10.3897/BDJ.13.e122993

**Published:** 2025-03-25

**Authors:** Jacky S Chitty, Daniel P Duran

**Affiliations:** 1 Rowan University, Glassboro, United States of America Rowan University Glassboro United States of America

**Keywords:** Chalcidoidea, Monodontomerini, parasitoid, Saint Anthony’s Dunes, species description, taxonomy

## Abstract

**Background:**

The genus *Monodontomerus* Westwood, 1833 (Hymenoptera, Torymidae) is distributed throughout the Globe, mostly in the Holarctic Region. Members of this genus are prolific parasitoids of pollinators and some are economically important. The New World fauna includes 25 described species and was revised 25 years ago.

**New information:**

Two new species of parasitoid wasp, *Monodontomerusrhinokopia* Chitty and Duran **sp. nov.** and *Monodontomerusverdigris* Chitty and Duran **sp. nov.** (Torymidae, Monodontomerinae) are described from the Pacific Northwest region of North America. *Monodontomerusrhinokopia* may be differentiated from other species in the genus by simultaneously possessing the following combination of characteristics: a face not bulging in profile, malar sulcus well defined and straight (Fig. 5), F1-F7 subquadrate (Fig. 7), longitudinal irregular carinae on its dorsellum (Fig. 8), sculpture of mesepimeron confined to ventral margin (Fig. 5), apical rim of scutellum produced posteriorly and not emarginate, costal cell on anterior margin above with row in distal half and lacking setae basally, dorsal admarginal setae reaching both marginal vein and parastigma (Fig. 6) and Mt1 reticulately sculptured dorsally. *Monodontomerusverdigris* may be differentiated from other members of the genus by simultaneously possessing the following characteristics: F1-F7 subquadrate (Fig. 3), striation in the anterodorsal corner of the mesepimeron which does not reach transepimeral sulcus (Fig. 1), carina of dorsellum which splits into two distinct carinae that form an open “V” (Fig. 4), median depression of propodeum narrowly triangular to nucha which it intercepts with nearly parallel lateral margins, a projecting rim of the scutellum which is not emarginated and a reticulately textured Mt1 (Fig. 2). A revised key to the New World species of *Monodontomerus* has been created to enable identification of these species.

## Introduction

Approximately two million species of organisms have been described as of the present (IUCN 2023), but likely about 8.7 million (+/- 1.3 million) species exist on Earth (Mora et al. 2011). Given that so many species remain unknown and undescribed, this “taxonomic impediment” is a serious concern to the preservation of our planet’s rapidly diminishing biodiversity (Wheeler et al. 2004). Moreover, the groups of insects that dominate species diversity are the ones that have been the most severely neglected by taxonomists (Srivathsan et al. 2023). Recent estimates of species richness have revealed the Hymenoptera (bees, wasps and ants) to be the most speciose order, but it greatly lags behind Coleoptera (beetles) in number of described species (Forbes et al. 2018).

One of the many genera belonging to the order Hymenoptera is *Monodontomerus* Westwood, 1833, part of the family Torymidae (Janšta et al. 2017). Here, we contribute two species to the known diversity of this genus in the New World, previously with 25 species described from this biogeographic region. Many members of this genus are prolific parasitoids of pollinators (Zerova and Seregina 2002) and some are economically important (Grissell 2000).

The type localities of both new species are in the Pacific Northwest, where 29% of North America’s insect biodiversity can be found. The most common type of habitat in this region is evergreen forests, which usually receive comparatively high levels of rainfall and are composed of western coastal tree species. However significant variability in elevation and available water in this region leads to a wide range of ecosystems being found here, such as meadows, deserts and alpine habitats (Danks 1995).

## Materials and methods

Surveys of parasitic wasps at St. Anthony’s Sand Dunes (Bureau of Land Management) in south-eastern Idaho, USA were conducted in the summer of 2022 by Ross Winton, Stephen Roman, Dan Taylor and the second author. Specimens were collected using malaise traps and V-FIT traps. During these surveys a lone specimen of a female *Monodontomerus* species was collected between 26 June and 28 June 2022. Upon examination of the specimen under a microscope, the first author believed it to be an undescribed species. While examining unidentified *Monodontomerus* specimens at the National Museum of Natural History, the first author found three specimens which also represent a new species. These specimens were collected by R.P. Curie in Kaslo, British Columbia, Canada.

The morphological terminology in this paper follows Grissell (2000). The specimen was compared to all species descriptions from Grissell’s revision of the genus in the New World, as well as holotype or lectotype specimen photos of the putative closest relatives, including *M.dentipes* (Dalman, 1820) *and M. minor* (Ratzeburg, 1848). The holotype of *M.brevicrus* (Grissell, 2000), *M.dianthidii* (Gahan, 1941) and *M.tectus* (Grissell, 2000) were examined in person at the National Museum of Natural History

Images of the frontal, lateral and dorsal habitus were taken using a Canon EOS 6D camera and were processed using ZereneStacker (Build T2023-06-11-1120) and Adobe Photoshop 2023. Propodeum images were taken using a Canon EOS 6D Mark II camera and processed using Helicon Focus. Measurements were taken by analysing photos using ImageJ. The holotype photos for *Monodontomerusrhinokopia* **sp. nov.** used the following magnifications: 20x for the frontal photo, 5x for the dorsal and lateral and 10x for the propodeum. The male paratype photos for *M.rhinokopia* **sp. nov.** used the following magnifications: 20x for the frontal photo and 7.5x for the dorsal and lateral. The holotype photos for *Monodontomerusverdigris* **sp. nov.** used the following magnifications: 20x for the frontal photo, 5x for the dorsal and lateral and 10x for the propodeum.

All type material is deposited in the National Museum of Natural History (Smithsonian) in Washington DC (address: 10^th^ St. & Constitution Ave. NW, Washington, DC 20560).

## Taxon treatments

### Monodontomerus verdigris

Chitty & Duran sp. nov.

443B59D6-EE70-52C5-94E0-267FBAD2A811

D1406573-956C-4B67-9AFC-C87E5E421843

#### Materials

- **Type status:** Holotype. **Occurrence:** individualCount: 1; sex: female; occurrenceID: 00D62C41-F3DB-5741-9AC7-3C205462B72D; **Location:** higherGeography: North America; country: United States of America; stateProvince: Idaho; county: Fremont; locality: Saint Anthony’s Dunes; verbatimLocality: USA ID: Fremont Co. / Saint Anthony’s Dunes / 44.117199, -111.650936; verbatimLatitude: 44.117199; verbatimLongitude: -111.650936; **Event:** samplingProtocol: V-shaped flight intercept trap; eventDate: 2022-06-26 to 2022-06-28; verbatimEventDate: 26-28 June 2022; habitat: sand dune; **Record Level:** institutionID: http://grscicoll.org/institution/smithsonian-institution-national-museum-natural-history; institutionCode: NMNH; basisOfRecord: PreservedSpecimen

#### Description

Figs 1, 2, 3, 4

Body length 3.7 mm, excluding ovipositor (5.0 mm with ovipositor). Colour metallic greenish-black and, under certain lighting, with shining bluish-green highlights, especially on hind coxae and femora; scape, apices of pro- and mesofemur, tibia and tarsi pale testaceous. Pedicel black and with green reflections under certain lighting, flagellum black. Apices of tarsi black.

Head: Face transverse in outline, width:height ratio 5:4; clypeus lying within imaginary line drawn between lateral corners of oral fossa; intermalar distance about 4.0x malar distance; malar sulcus present; lower face not bulging in profile; toruli about own diameter above ventral margin of eye; eye with setae, shorter than those on genae (Fig. 3).

Antenna: Antennal formula is 11173 and typical of genus, scape not reaching mid-ocellus, about 3x length of pedicel; funicular segments subquadrate (Fig. 3).

**Figure 1.**
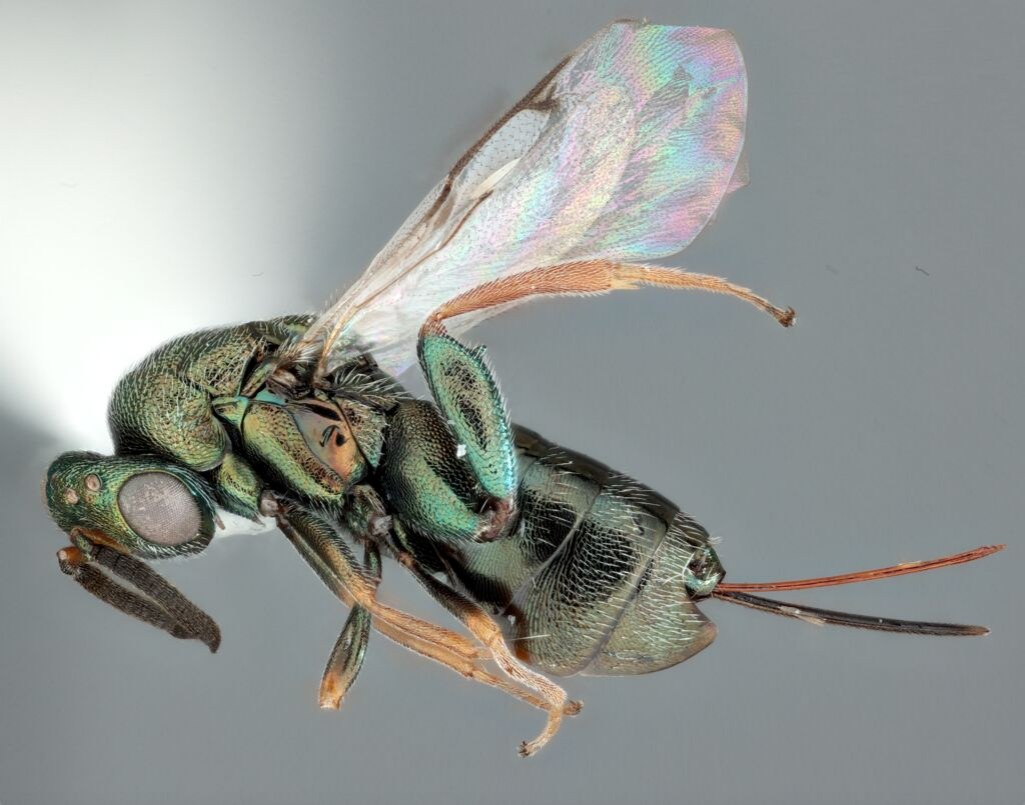
Lateral habitus of *Monodontomerusverdigris* Chitty & Duran sp. nov. holotype.

**Figure 2.**
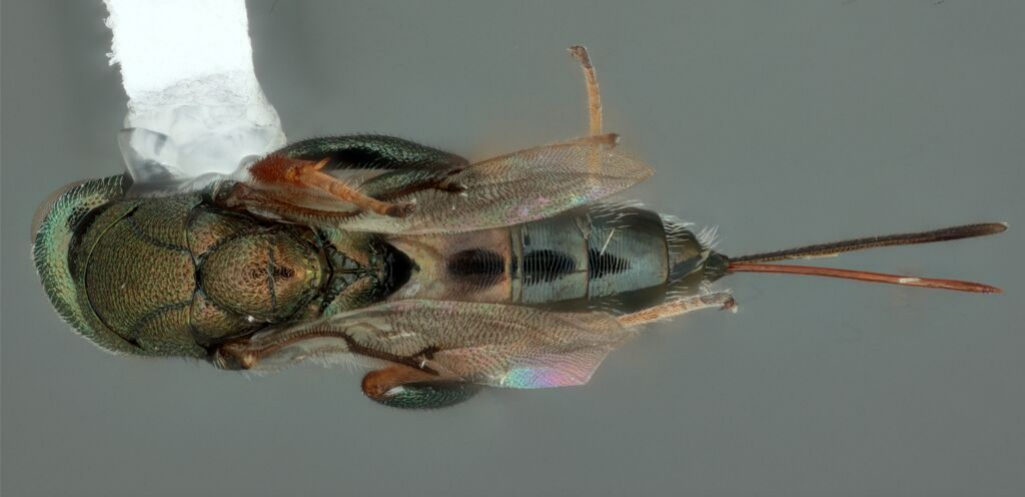
Dorsal habitus of *Monodontomerusverdigris* Chitty & Duran sp. nov. holotype.

Mesosoma: Mesepimeron highly polished overall, except for sculpture above ventral margin and striation on anterodorsal margin, striation of anterodorsal mesepimeron not reaching transepimeral sulcus, transepimeral sulcus not extending to ventral margin (Fig. 1); frenal area about 0.4x length of scutellum, sculptured overall, apical rim produced posteriorly with punctures larger at apex, not emarginate (Fig. 2); dorsellum medially carinate, dorsally splits into two distinct carinae that form an open V, scarcely projecting ventrally as tooth, surface lateral to carina convex, with distinct pits on dorsal margin of dorsellum, pits do not extend down to ventral margin along sides of carinae (Fig. 4); propodeum with median depression triangular and extending to nucha, lateral margins converging strongly, lateral areas with reticulate sculpture; median carina dorsally slightly divided (Fig. 2).

Forewing: Costal cell on anterior margin above with setal row in distal half and lacking setae proximally, below with two complete apical setal rows and additional setae in distal 1/3, cubital and basal veins with setae; basal cell with setal row, dorsal admarginal setae reaching marginal vein, but not parastigma, discal setae extending to mid-point of basal area; stigmal area faintly stained around stigma, stigma and uncus elongate; marginal vein about 0.4x length of costal cell, post marginal vein about 0.6x length of marginal vein, distal portion of postmarginal vein subequal to length of proximal portion (Fig. 1).

Leg: Metacoxa width is about 0.6x its height; hind femur about 3x as long as wide; hind femoral tooth as in Fig. 1; tibia and femur subequal in length, longest hind tibial spur about 0.33x length of basitarsus (Fig. 1).

Metasoma: Mt1 reticulately sculptured; Mt5 acutely curved in profile; ovipositor 0.4x length of body (Fig. 1)

LATERAL (Fig. 1) Measurements – 669 pixels per 1 mm

All dimensions in mm.

Body length – 3.709 mm, Ovipositor length – 1.554, Mesepimeron height – 0.515, Mesepimeron width – 0.238, Mesepisternum height – 0.661, Mesepisternum width – 0.269, Postmarginal vein length – 0.334, Marginal vein length – 0.534, Costal cell length – 1.232, Meta femur length – 1.082, Metafemur width – 0.323, Metacoxae height – 0.904, Metacoxa width – 0.469, Meta tibia length – 0.972, Metatarsi length – 0.972, Basitarsus length – 0.313, Metatibial spur length – 0.137, Flagellum length – 0.828, Pedicel length – 0.131, Eye height– 0.513.

DORSAL (Fig. 2) Measurements – 669 pixels per 1 mm.

Scutellum length – 0.691 Frenal area length– 0.274.

**Figure 3.**
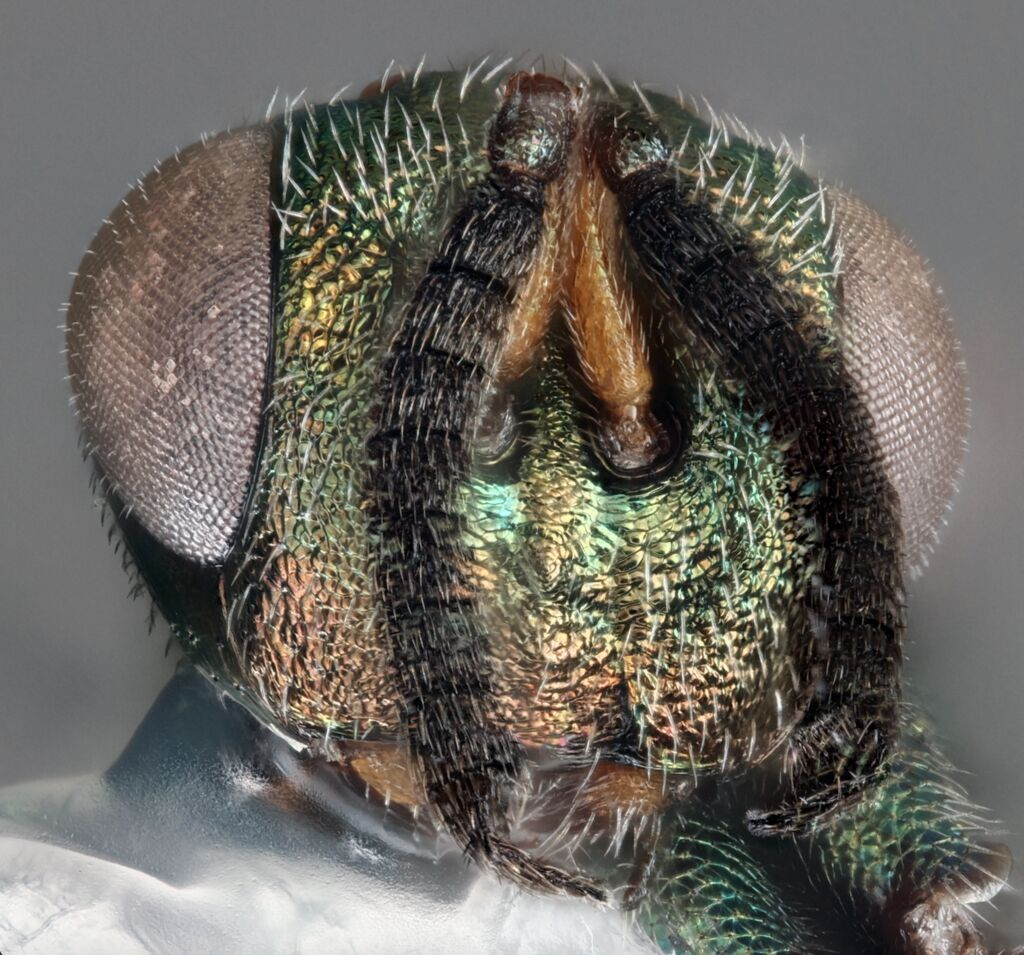
Anterior habitus of *Monodontomerusverdigris* Chitty & Duran sp. nov. holotype.

ANTERIOR (Fig. 3) Measurements – 1190.966 pixels per 1 mm.

Malar distance – 0.168 Intermalar distance – 0.789 Scape – 0.323 Pedicel – 0.111.

#### Diagnosis

**Figure 4.**
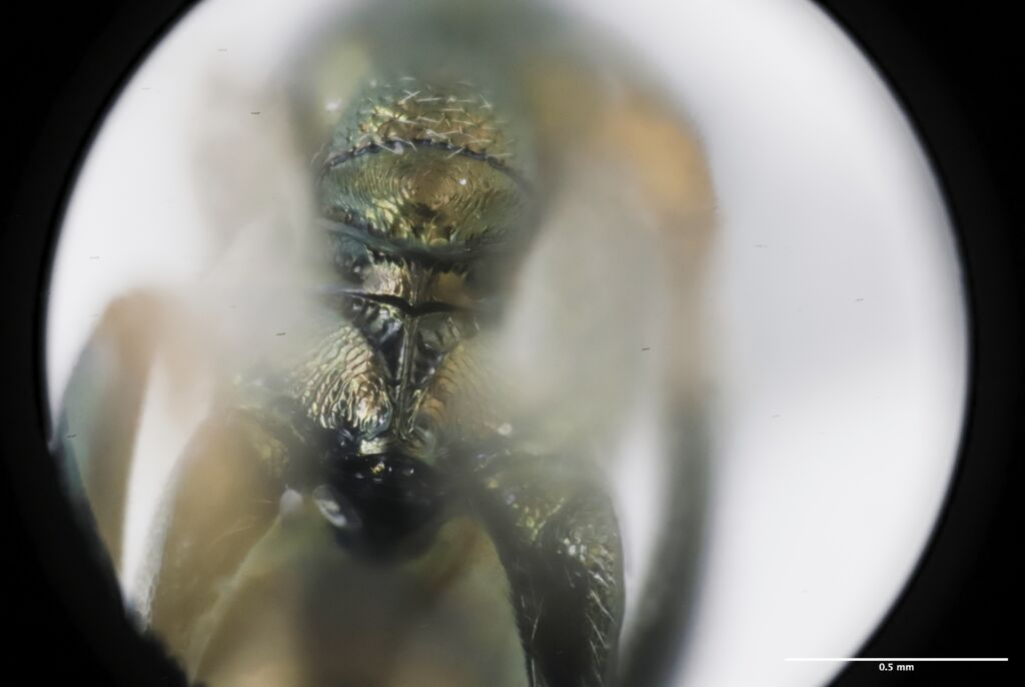
Propodeum habitus of *Monodontomerusverdigris* Chitty & Duran sp. nov. holotype.

*Monodontomerusverdigris* **sp. nov.** (Figs 1, 2, 3, 4) can be distinguished from all other species in the genus by the following combination of characteristics: F1-F7 subquadrate (Fig. 3), striation in the anterodorsal corner of the mesepimeron which does not reach transepimeral sulcus (Fig. 1), carina of dorsellum which splits into two distinct carinae that form an open "V" (Fig. 4), median depression of propodeum narrowly triangular to nucha which it intercepts with nearly parallel lateral margins, a projecting rim of the scutellum which is not emarginated and a reticulately textured Mt1 (Fig. 2).

It can be distinguished from *Monodontomerusbrevicrus* by possessing the following combination of characteristics: Intermalar distance 4x malar distance, F1-F7 subquadrate (Fig. 3), possessing a diagonal band of sculpture in anterodorsal corner of mesepimeron, transempimeral sulcus not extending to ventral margin (Fig. 1), apical rim conspicuously produced posteriorly and with punctures of rim of scutellum wider at apex (Fig. 2), median carina of dorsellum dorsally splitting into two distinct carinae that form an open V (Fig. 4), median carina of dorsellum not subtended by grooves (Fig. 4), strongly defined median carina of propodeum (Fig. 2), stigmal area faintly stained only immediately around stigma, distal portion of post marginal vein are subequal to proximal portion instead of two-thirds its length and Mt5 acutely curved in profile (Fig. 1).

It can be distinguished from *Monodontomerusdianthidii* by possessing the following combination of characteristics: greenish-black body, intermalar distance 4x malar distance, F1-F7 subquadrate (Fig. 3), possessing a diagonal band of sculpture in anterodorsal corner of mesepimeron (Fig. 1), frenal area not polished under any lighting, apical rim of scutellum is not emarginate (Fig. 2), carina of dorsellum dorsally splitting into carinae that form an open V (Fig. 1), lacking setae basally on anterior margin above of costal cell, admarginal setae reaching marginal vein, but not parastigma and hind femoral tooth more elongate (see Fig. 1 and fig. 37 from Grissell (2000)).

It can be distinguished from *Monodontomerusdentipes* by possessing the following combination of characteristics: intermalar distance about 4x malar distance, setae covering eyes not denser than those of genae or head, F1-F7 subquadrate, toruli only 1x own diameter above ventral margin of eye (Fig. 3), sculpture on anterodorsal margin of upper mesepimeron not extending to transepimeral sulcus (Fig. 1), frenal area sculptured throughout (Fig. 2), median carina of dorsellum dorsally splitting into two distinct carinae that form an open V, dorsal margin of dorsellum not lacking pits, area lateral to carina of dorsellum convex instead of flat or concave, tooth of dorsellum not projecting into carina of propodeum, median carina of propodeum not dorsally greatly forked (Fig. 4), costal cell on anterior margin above with setal row only in distal half, costal cell on anterior margin below lacking additional setae in basal fifth, discal setae not extending into basal area as parallel rows, hind femoral tooth clearly more than own length from apex and Mt5 acutely curved in profile (Fig. 1).

It can be distinguished from *Monodontomerusminor* by possessing the following combination of characteristics: intermalar distance about 4x malar distance, toruli only 1x own diameter above ventral margin of eye, F1-F7 subquadrate (Fig. 3), possessing a diagonal band of sculpture in anterodorsal corner of mesepimeron (Fig. 1), frenal area not polished, apical rim of scutellum not interrupted posteriorly at median margin by polished area (Fig. 2), surface lateral to carina of dorsellum not concave, tooth of dorsellum not projecting into carina of propodeum (Fig. 4), median carina of propodeum not deeply V-shaped (Fig. 1), costal cell on anterior margin above with setal row only in distal half and also lacking additional row in apical third, costal cell below not with extra setae in basal fifth, discal setae not extending into basal area as parallel rows, stigmal area faintly stained around stigma and not any further, hind femoral tooth is shorter and projecting straight (not at 45 degree angle, see Fig. 1 and fig. 19 from Grissell (2000)), Mt1 reticulately sculptured and Mt5 acutely curved in profile (Fig. 1).

It can be distinguished from *Monodontomerustectus* by possessing the following combination of characteristics: F3-7 subquadrate (Fig. 3), striation in anterodorsal corner of mesepimeron not reaching transepimeral sulcus (Fig. 1), carina of dorsellum dorsally splitting into carinae that form an open V (Fig. 4), carina of dorsellum projecting ventrally as tooth, median depression of propodeum narrowly triangular to the nucha, which it intercepts with strongly convergent lateral margins, transverse carinae of propodeum not as heavy and median carina of propodeum dorsally lacking distinct V-shaped raised area composed of fine longitudinal carinae (Fig. 2).

In addition, see modified key couplets of Grissell (2000) below.

#### Etymology

*Monodontomerusverdigris* is named for the colour of the hind coxae and femora which possess bright bluish-green reflections. Verdigris is the word for copper salts that commonly form on copper statues, derived from Old French (“verte grez” for green-grey). The word verdigris also starts with "V" and a "V"-shaped structure on the dorsellum which is one of the species' defining features.

#### Distribution

Little is known about this species, as it is based on a single specimen, not observed in life and collected via a passive trapping device, a V-FIT (i.e. “V”-shaped Flight Intercept Trap). Given that the habitat of the St. Anthony’s Sand Dunes is a unique section of the Greater Yellowstone Ecosystem comprised of large aeolian sand dunes and short vegetation, it is likely to be a specialist on a sand-dwelling host species.

### Monodontomerus rhinokopia

Chitty & Duran sp. nov.

F0582EF1-2603-56BF-A1BE-D77CF0E37E73

CABC6894-8D11-4B90-9C6A-D26D8752B390

#### Materials

- **Type status:** Holotype. **Occurrence:** individualCount: 1; sex: female; lifeStage: adult; occurrenceID: 30678388-0D4C-55DB-96A7-15A1E3970644; **Location:** higherGeographyID: North America; country: Canada; stateProvince: British Columbia; locality: Kaslo; verbatimLocality: Kaslo / 2 VII 03 BC; **Event:** samplingProtocol: unknown; verbatimEventDate: 2 VII 03; **Record Level:** institutionID: http://grscicoll.org/institution/smithsonian-institution-national-museum-natural-history; institutionCode: NMNH; basisOfRecord: PreservedSpecimen
- **Type status:** Paratype. **Occurrence:** individualCount: 1; sex: male; lifeStage: adult; occurrenceID: 822A10B4-2C76-5314-971C-9322ED6027EE; **Location:** higherGeographyID: North America; country: Canada; stateProvince: British Columbia; locality: Kaslo; verbatimLocality: Kaslo / 2 VII 03 BC; **Event:** samplingProtocol: unknown; verbatimEventDate: 2 VII 03; **Record Level:** institutionID: http://grscicoll.org/institution/smithsonian-institution-national-museum-natural-history; institutionCode: NMNH; basisOfRecord: PreservedSpecimen
- **Type status:** Paratype. **Occurrence:** individualCount: 2; sex: female; lifeStage: adult; occurrenceID: F1CF48C5-AF55-5539-B9D6-D664F6453E57; **Location:** higherGeographyID: North America; country: Canada; stateProvince: British Columbia; locality: Kaslo; verbatimLocality: Kaslo / 2 VII 03 BC; **Event:** samplingProtocol: unknown; verbatimEventDate: 2 VII 03; **Record Level:** institutionID: http://grscicoll.org/institution/smithsonian-institution-national-museum-natural-history; institutionCode: NMNH; basisOfRecord: PreservedSpecimen

#### Description

Figures

**Figure 5.**
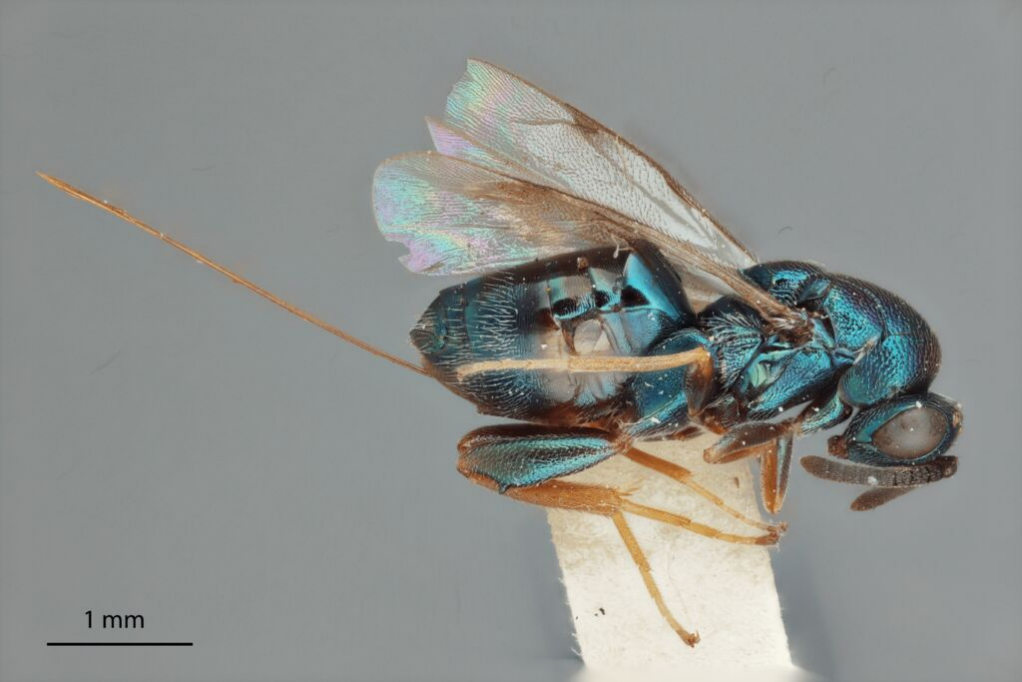
Lateral habitus of *Monodontomerusrhinokopia* Chitty & Duran sp. nov. holotype.

**Figure 6.**
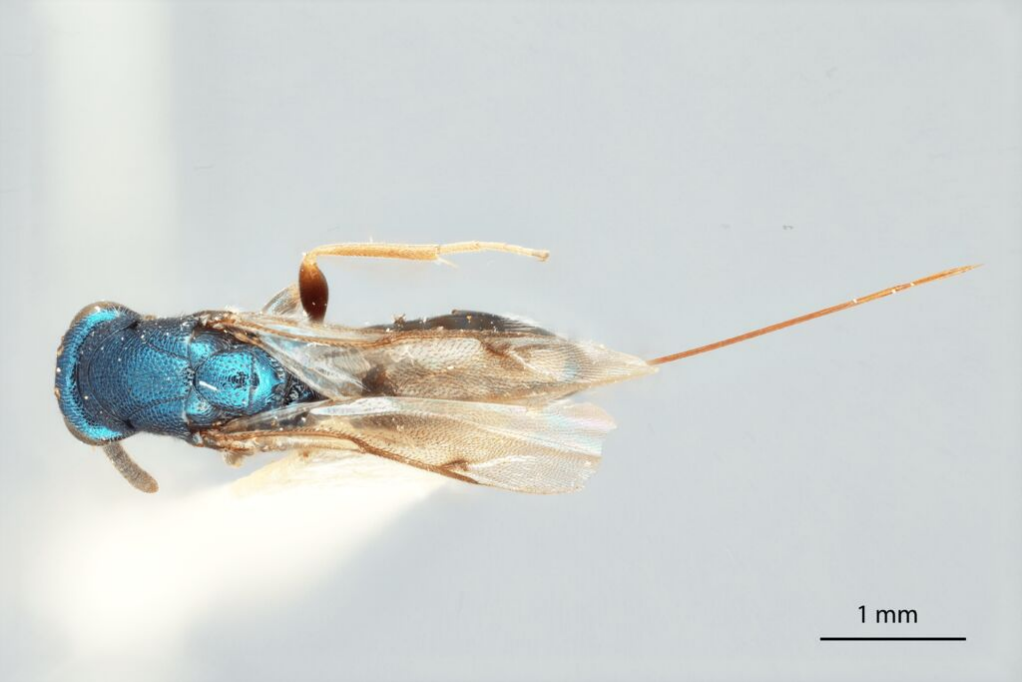
Dorsal habitus of *Monodontomerusrhinokopia* Chitty & Duran sp. nov. holotype.

**Figure 7.**
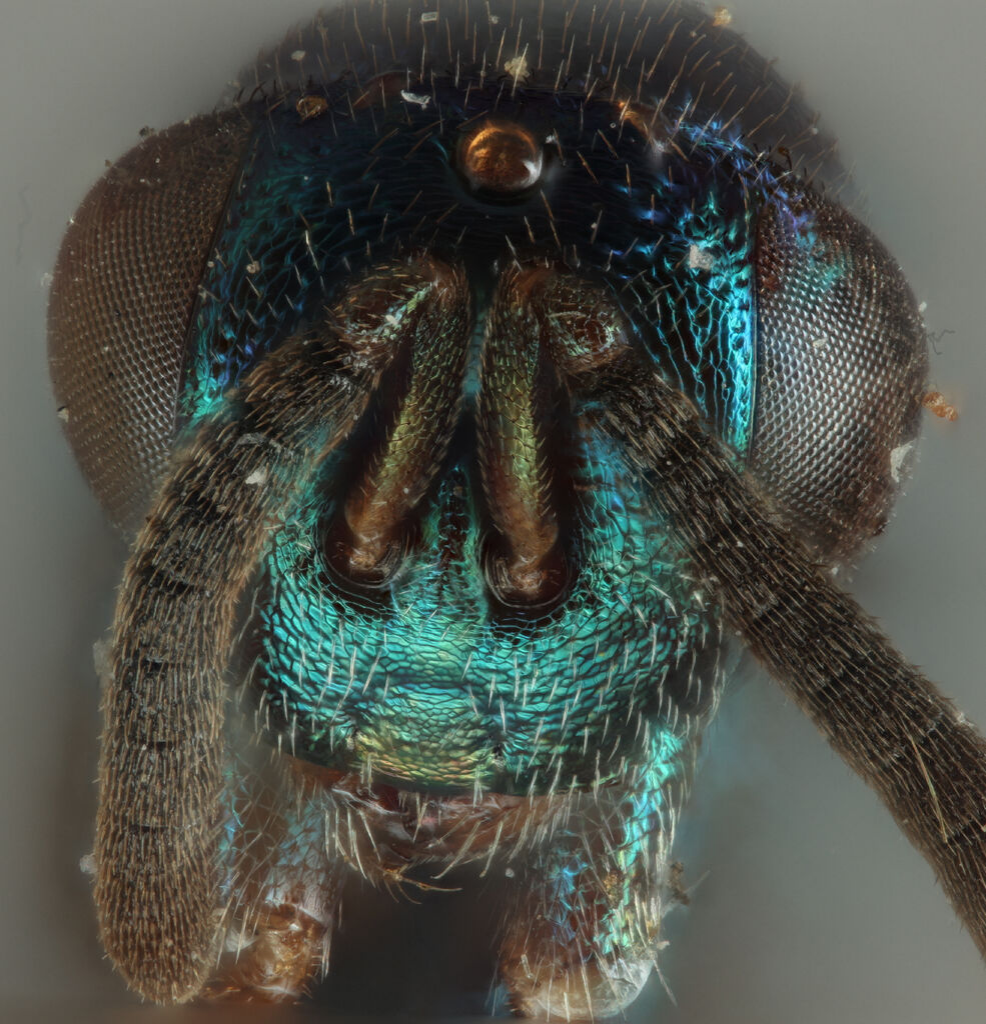
Frontal habitus of *Monodontomerusrhinokopia* Chitty & Duran sp. nov. holotype.

**Figure 8.**
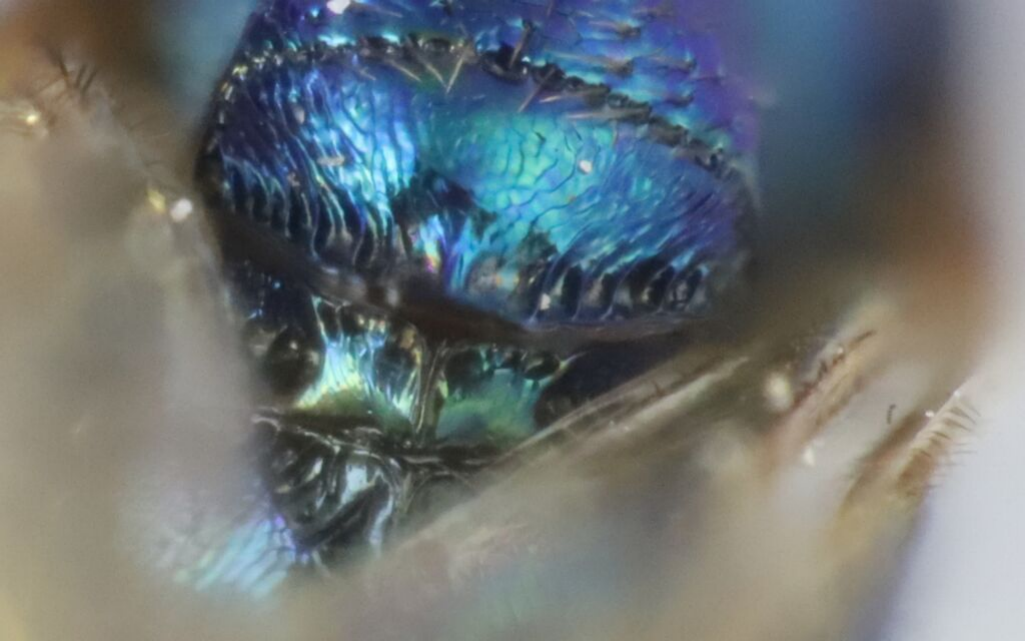
Propodeum habitus of *Monodontomerusrhinokopia* Chitty & Duran sp. nov. female paratype.

**Figure 9.**
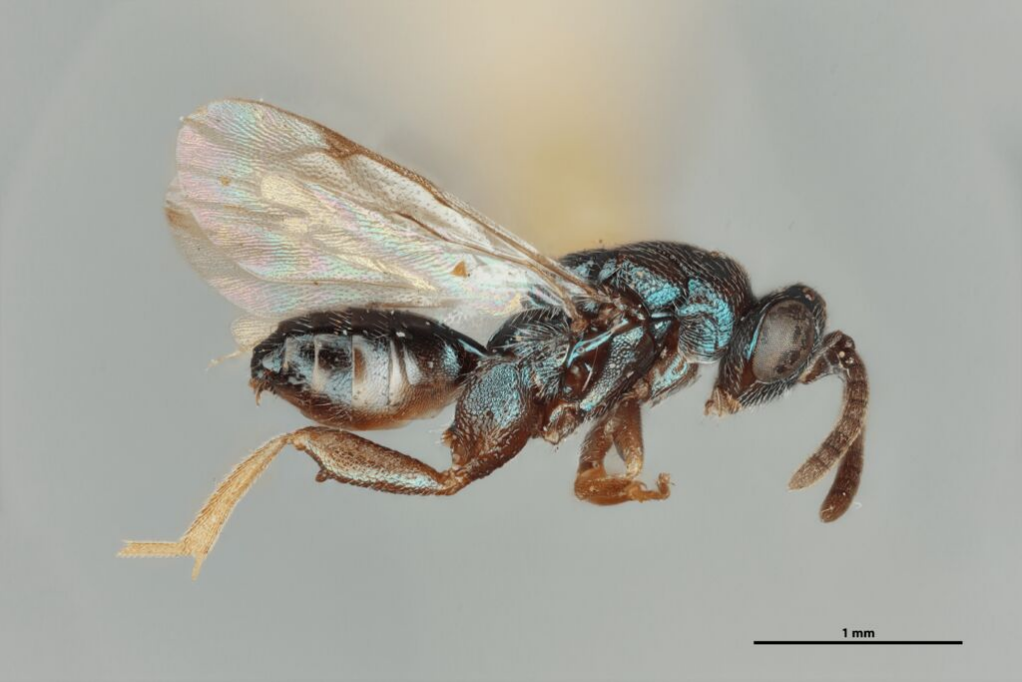
Lateral habitus of *Monodontomerusrhinokopia* Chitty & Duran sp. nov. paratype (male).

**Figure 10.**
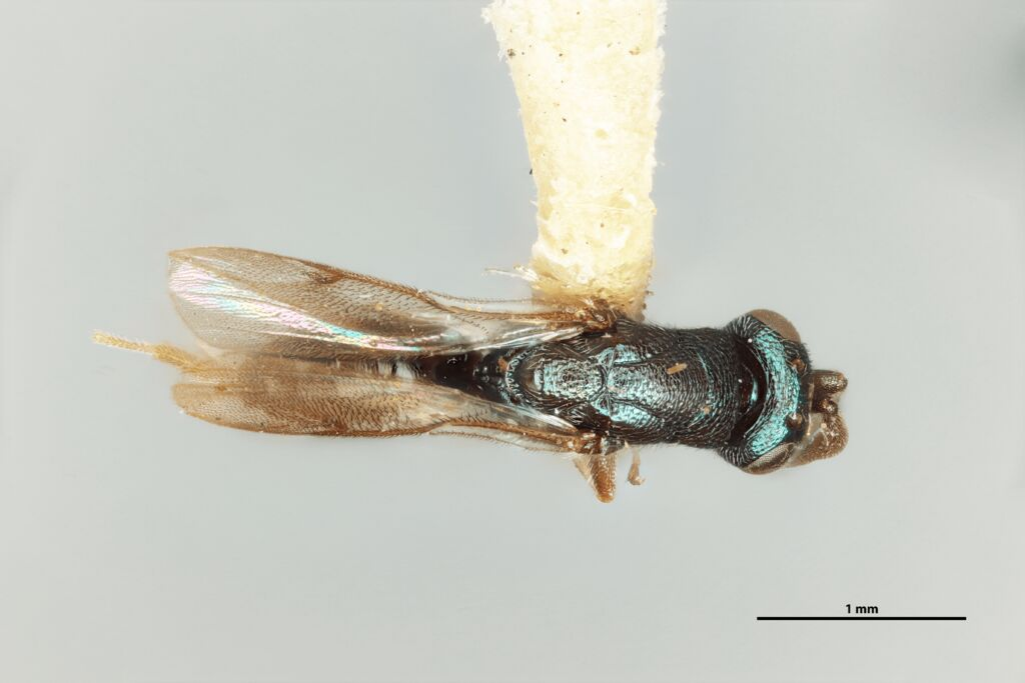
Dorsal habitus of *Monodontomerusrhinokopia* Chitty & Duran sp. nov. paratype (male).

**Figure 11.**
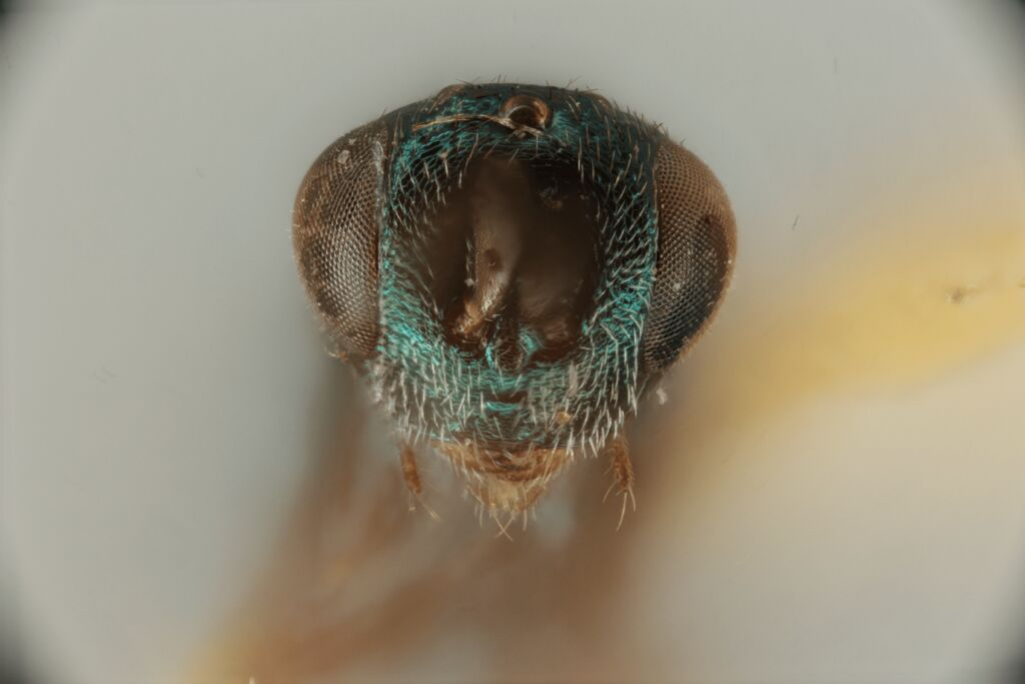
Frontal habitus of *Monodontomerusrhinokopia* Chitty & Duran sp. nov. paratype (male).

Figs 5, 6, 7, 8, 9, 10, 11

Body length 3.9 mm, excluding ovipositor (6.9 mm including ovipositor). Colour metallic-blue body, coxae and femora; scape infused metallically distally; tibia and tarsi pale testaceous.

Head: Width height ratio 5:4, clypeus lying within imaginary line drawn between lateral corners of oral fossa; intermalar distance about 2x malar distance; malar sulcus present, strongly developed and straight; mandibles each with 3 teeth; toruli about own diameter above ventral margin of eye; lower face not bulging in profile; eye appearing almost asetose, but with spare setae not as long or as dense as on cheek or dorsum of head; scape not reaching midocellus, about 3x length of pedicel (Fig. 7).

Antenna: Antennal formula is 11173 and typical of genus, F1-F7 subquadrate (Fig. 7).

Mesosoma: Mesepimeron appearing highly polished overall with sculpturing above ventral margin, transepimeral sulcus incomplete (Fig. 5); frenal area 0.3x length of scutellum, appearing polished overall, but faint alutaceous sculpture may be seen medially in some lighting and sculpture more pronounced laterally, apical rim of scutellum produced posteriorly with punctures wider at apex than laterally (Fig. 6), dorsellum with longitudinal irregular carinae (Fig. 8), ventral margin slightly produced medially as tooth subtended by pits, pits of dorsal margin extending beyond dorsolateral corners, pits of dorsal margin not extending along median carina to ventral margin (Fig. 8); propodeum with median depression triangular and extending almost to nucha, lateral margins converging strongly, lateral areas strongly reticulate, median carina of propodeum not dorsally divided (Fig. 8).

Forewing: Costal cell on anterior margin above with setal row in distal half, costal cell on anterior margin below with two complete setal rows and additional setae in proximal 1/5 and distal 1/3, cubital and basal vein setose, basal cell with partial setal row, dorsal admarginal setae reaching to marginal vein and parastigma, stigma and uncus elongate, marginal vein about 0.5x length of costal cell, post marginal vein about 0.5x length of marginal vein, distal portion of post marginal vein is subequal to its proximal portion (Fig. 6).

Leg: Metacoxa width is about 0.6x its height; hind femur about 4x as long as wide, hind femoral tooth as seen in Figure 5; length of femur and tibia subequal (Fig. 5).

Metasoma: Mt1 completely sculptured dorsally; Mt5 acutely curved in profile; ovipositor 1.0x length of body (Fig. 5).

Male: Body length 2.8 mm, scape metallic-green, in profile slightly curved and of about even thickness; venter of scape a flat, curved, asetose and highly polished area; foreleg unmodified (Fig. 9).

LATERAL (Fig. 5) Measurements – 1 mm every 768 pixels.

All dimensions in mm.

Body length – 3.068, Ovipositor length – 3.068, Mesepimeron height – 0.519, Mesepimeron width – 0.215, Mesepisternum height – 0.711, Mesepisternum width – 0.256, Postmarginal vein length – 0.260, Marginal vein length – 0.525, Costal cell length – 1.217, Metafemur length – 1.167, Metafemur height – 0.297, Metacoxae height – 0.862, Metacoxae width – 0.526, Metatibia length – 1.039, Metatarsi length – 1.144, Flagellum length – 0.109, Pedicel length – 0.099, Eye height – 0.527.

DORSAL (Fig. 6) Measurements – 1 mm every 756 pixels.

Scutellum length – 0.649, Frenal area length– 0.211.

ANTERIOR (Fig. 7) Measurements – 0.5 mm every 1470 pixels.

Malar distance – 0.199 Intermalar distance – 0.662 Scape – 0.321 Pedicel – 0.110.

LATERAL MALE (Fig. 9) Measurements - 1 mm every 1098 pixels.

Body length – 2.815, Metasoma - 1.113, Metafemur length – 0.804, Metafemur width – 0.211, Head Length – 0.661, Postmarginal vein length – 0.200, Marginal vein length – 0.369, Costal cell length – 0.984, F7 length – 0.085, F7 width – 0.102, Scape length – 0.283.

**Variation**

The female paratype and particularly the male paratype have somewhat less irregular dorsellum carinae than the holotype. A species which also possessed irregular dorsellum carinae, *Monodontomerusbakeri*, is mentioned in Grissell (2000) to have some variation in its dorsellum, based on size. This possibly could be the case in this species as well, given the male’s smaller body.

The medial scutellum of the male paratype appears polished under all lighting, with sculpture laterally, whereas both the holotype and female paratype appear medially sculptured under certain lighting. It is also unknown whether this represents variation or sexual dimorphism, given that only one male is known.

While still incomplete in the female paratype, the transepimeral sulcus can very faintly be traced to the ventral margin under some lighting, but no groove is present.

#### Diagnosis

*Monodontomerusrhinokopia* **sp. nov.** (Figs 5, 6, 7, 8, 9, 10, 11) can be distinguished from all other species in the genus by the following combination of characteristics: face not bulging in profile, malar sulcus well defined and straight (Fig. 5), F1-F7 subquadrate (Fig. 7), longitudinal irregular carinae on its dorsellum (Fig. 8), sculpture of mesepimeron confined to ventral margin (Fig. 5), apical rim of scutellum produced posteriorly and not emarginate, costal cell on anterior margin above with row in distal half and lacking setae basally, dorsal admarginal setae reaching both marginal vein and parastigma (Fig. 6) and Mt1 reticulately sculptured dorsally.

It can be distinguished from *M.bakeri* by possessing the following combination of characteristics: malar sulcus strongly defined and straight, toruli about own diameter above ventral margin of eye (Fig. 7), lower face not bulging in profile (Fig. 5), apical rim of scutellum produced posteriorly with punctures wider at apex than laterally, pits of dorsal margin extending beyond dorsolateral corners (Fig. 8), costal cell on anterior margin above with setal row in distal half, costal cell on anterior margin below with two complete setal rows and additional setae in proximal and distal 1/3, dorsal admarginal setae reaching to marginal vein and parastigma, Mt1 completely sculptured dorsally (Fig. 6); Mt5 acutely curved in profile (Fig. 5).

It can be distinguished from *M.clementi* by possessing the following combination of characteristics: clypeus lying within imaginary line between oral fossa and not protruding (Fig. 7), sculpture of mesepimeron confined to ventral margin, groove of transepimeral sulcus not extending to ventral margin (Fig. 5), frenal area either polished or polished at some angles, costal cell on anterior margin above with row in distal half and lacking setae basally, dorsal admarginal setae reaching both marginal vein and parastigma (Fig. 6), Mt1 not polished anteromedially.

It can be distinguished from *M.dianthidii* by possessing the following combination of characteristics: colour steely blue-black body, F1-F7 subquadrate instead of wider than long (Fig. 7), apical rim of scutellum not emarginate, median carina of propodeum not dorsally divided (Fig. 8), additional setae found in proximal 1/3 on anterior margin costal cell below and the following male characteristics: area below toruli not bump-like and polished, setae in facial area unmodified (Fig. 11).

It can be distinguished from *M.tectus* by possessing the following combination of characteristics: colour steely blue-black body, F3-7 subquadrate instead of wider than long (Fig. 7), upper mesepimeron without sculpture in anterodorsal corner (Fig. 5), dorsellum slightly projecting ventrally as tooth, lateral margins of propodeum converging strongly, median carina of propodeum lacking V-shaped raised area composed of fine longitudinal carinae (Fig. 8), costal cell below with additional setae in basal 1/3 (Fig. 6) and the following characteristics in males: scape green and lightly curved in profile (Fig. 9).

In addition, see modified key couplets of Grissell (2000) below.

#### Etymology

The name of *Monodontomerusrhinokopia* is derived from its lack of a “nose-like” bulging face, like that of a similar species *Monodontomerusbakeri*. "Rhinokopia", derived from the verb rhinokopeo, is the Greek word for a judicial punishment in which the nose is amputated; often applied to disposed emperors of the Byzantine Empire.

#### Distribution

Little is known about this species, as all known specimens have been collected from a single location and lack a host record. The type locality is Kaslo in British Colombia.

#### Taxon discussion

When a malar sulcus is present in *M.bakeri*, it is almost always greatly curved and poorly defined. Grissell (2000) examined over 200 specimens of *M.bakeri* and found only a single specimen with a well-developed malar sulcus, but it was also greatly curved and differed in no other respects from the species other than a yellow scape. *M.rhinokopia* shares none of the former defining traits of *M.emarginatus*, now synonymised with *M.bakeri*. (Grissell 2000).

As only one male is known from this species, further specimens would help elucidate what differences are simple variance versus sexual dimorphism.

## Identification Keys

### Updated key couplets from Grissell (2000)

**Table:** 

1	Median depression of propodeum narrowly triangular to nucha which it intercepts with strongly convergent lateral margins (see propodeum in Fig. 12), propodeum lacking distinct V-shaped raised area composed of fine longitudinal carinae, dorsellum projecting ventromedially as a small tooth	2
–	Median depression of propodeum broadly rounded to nucha which it intercepts with nearly parallel lateral margins (see propodeum in Fig. 13), propodeum dorsally with distinct V-shaped raised area composed of fine longitudinal carinae; dorsellum not projecting ventromedially as tooth	*M.tectus* Grissell
2	Body either light blue or dark steely-blue, anterodorsal corner of mesepimeron lacking striation, scutellum either polished medially (Fig. 10) or appearing polished at only some angles (Fig. 6), carina of dorsellum does not split into two and form an open V (Fig. 8)	3
–	Body greenish-black, blue colouration is limited to highlights, anterodorsal corner of mesepimeron with striation (Fig. 1), scutellum not polished medially at any angle (Fig. 2), carina of dorsellum dorsally splits into two distinct carinae that form an open V (Fig. 4)	*verdigris* Chitty and Duran **sp. nov.**
3	F1-F7 wider than long, apical rim of scutellum emarginate, median carina of propodeum dorsally divided, no setae additional to setal rows in basal ⅓ of costal cell below (Fig. 12). Male: Area below each torulus slightly elevated and bump-like, which are polished.	*M.dianthidii* Gahan
–	F1-F7 subquadrate (Fig. 7), apical rim of scutellum not emarginate, dorsellum with longitudinal irregular carinae (Fig. 8), median carina of propodeum not dorsally divided, additional setae in basal ⅓ of costal cell below (Fig. 6). Male: Area below each torulus unmodified, similar to rest of face (Fig. 11)	*M.rhinokopia* Chitty and Duran **sp. nov.**

Below are modified couplets (8 through 10) from Grissell (2000).

## Discussion

The Pacific Northwest region of North America may be undersampled, especially with respect to this group of wasps. The new species described above are only known from a small number of specimens. Additional sampling may yield more specimens or shed light on the ecologically relationships of these *Monodontomerus* species and their hosts.

## Supplementary Material

XML Treatment for Monodontomerus verdigris

XML Treatment for Monodontomerus rhinokopia

## Figures and Tables

**Figure 12.**
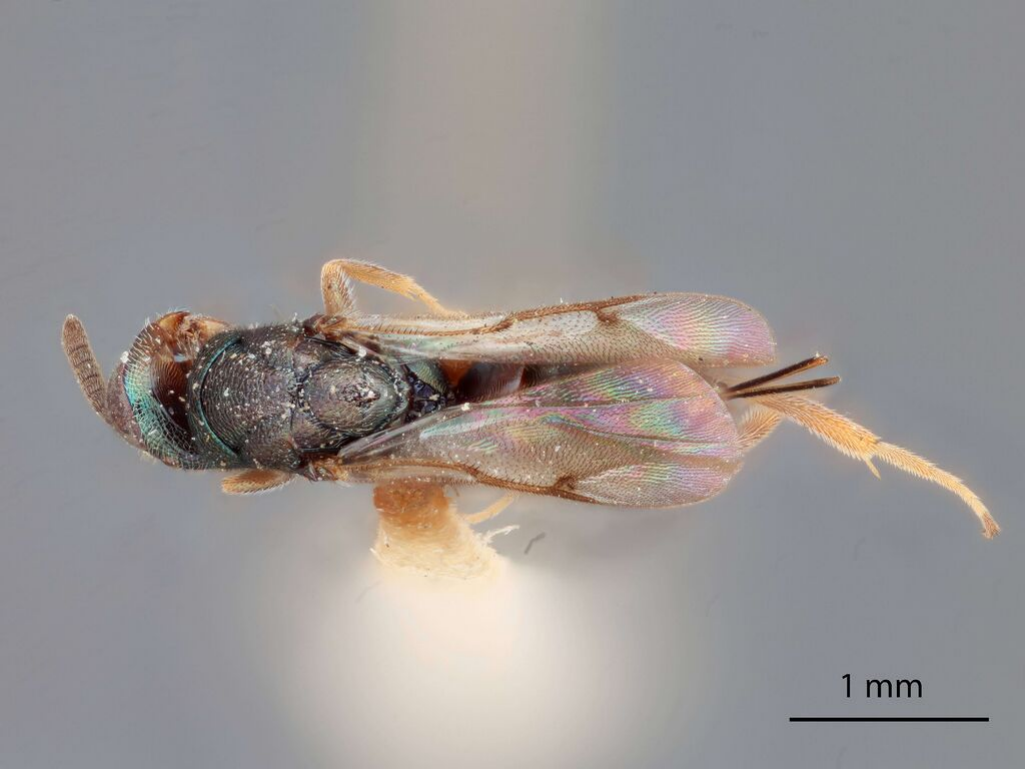
Dorsal habitus of *Monodontomerusdianthidii* Gahan 1941 holotype from Parr et al. (2014)

**Figure 13.**
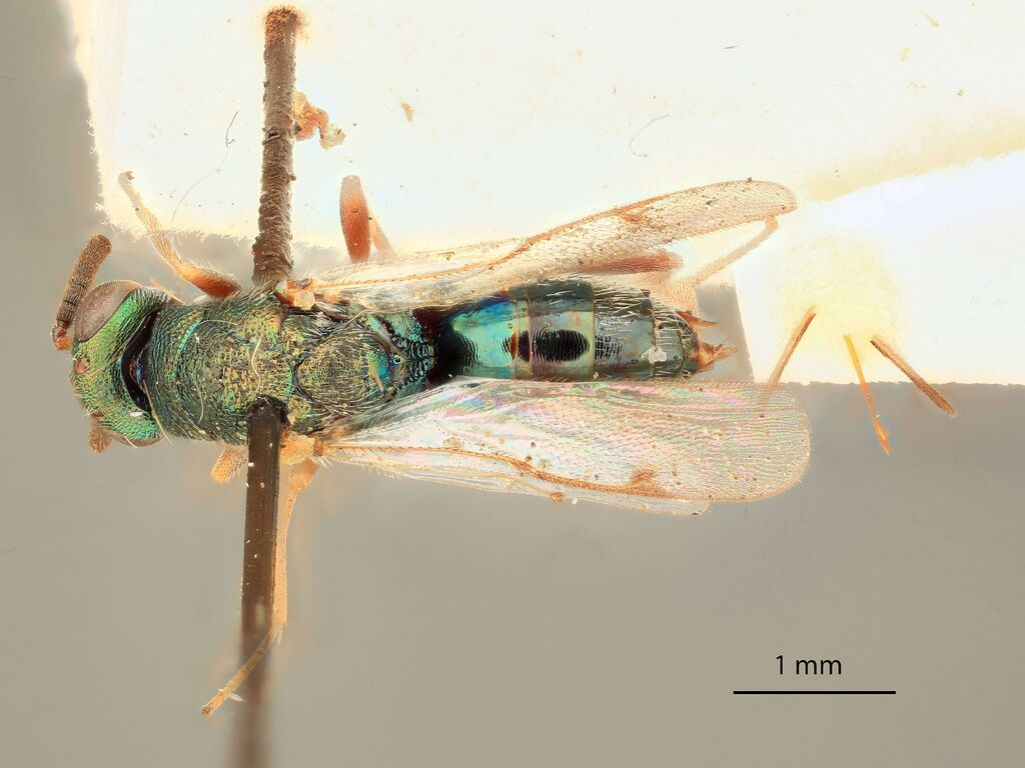
Dorsal habitus of *Monodontomerustectus* Grissell 2000 holotype from Parr et al. (2014)
